# Alterations of Lipid Metabolism in the Heart in Spontaneously Hypertensive Rats Precedes Left Ventricular Hypertrophy and Cardiac Dysfunction

**DOI:** 10.3390/cells11193032

**Published:** 2022-09-27

**Authors:** Tomasz K. Bednarski, Monika K. Duda, Pawel Dobrzyn

**Affiliations:** 1 Laboratory of Molecular Medical Biochemistry, Nencki Institute of Experimental Biology, Polish Academy of Sciences, 02-093 Warsaw, Poland; 2 Centre of Postgraduate Medical Education, Department of Clinical Physiology, 01-813 Warsaw, Poland

**Keywords:** SHR rats, lipogenesis, lipolysis, β-oxidation, hypertension, hypertrophy, myocardial metabolism

## Abstract

Disturbances in cardiac lipid metabolism are associated with the development of cardiac hypertrophy and heart failure. Spontaneously hypertensive rats (SHRs), a genetic model of primary hypertension and pathological left ventricular (LV) hypertrophy, have high levels of diacylglycerols in cardiomyocytes early in development. However, the exact effect of lipids and pathways that are involved in their metabolism on the development of cardiac dysfunction in SHRs is unknown. Therefore, we used SHRs and Wistar Kyoto (WKY) rats at 6 and 18 weeks of age to analyze the impact of perturbations of processes that are involved in lipid synthesis and degradation in the development of LV hypertrophy in SHRs with age. Triglyceride levels were higher, whereas free fatty acid (FA) content was lower in the LV in SHRs compared with WKY rats. The expression of de novo FA synthesis proteins was lower in cardiomyocytes in SHRs compared with corresponding WKY controls. The higher expression of genes that are involved in TG synthesis in 6-week-old SHRs may explain the higher TG content in these rats. Adenosine monophosphate-activated protein kinase phosphorylation and peroxisome proliferator-activated receptor α protein content were lower in cardiomyocytes in 18-week-old SHRs, suggesting a lower rate of β-oxidation. The decreased protein content of α/β-hydrolase domain-containing 5, adipose triglyceride lipase (ATGL) activator, and increased content of G0/G1 switch protein 2, ATGL inhibitor, indicating a lower rate of lipolysis in the heart in SHRs. In conclusion, the present study showed that the development of LV hypertrophy and myocardial dysfunction in SHRs is associated with triglyceride accumulation, attributable to a lower rate of lipolysis and β-oxidation in cardiomyocytes.

## 1. Introduction

Lipids and enzymes that are involved in their conversion are important regulators of cardiac metabolism and function. Triglycerides (TGs) that are stored within lipid droplets have no direct toxic effect on the myocardium [1], although TG accumulation in the myocardium is related to heart failure in obesity and diabetes mellitus [2]. It has been shown that TG are related to left ventricular (LV) mass in hypertensive patients [3] and TG content is significantly higher in the hypertensive heart disease than in hypertrophic cardiomyopathy [4]. In untreated hypertensive patients, high TG to glucose ratio correlated with left atrial volume index, LV mass index, E/e′ ratio, and e′ velocity [5]. Moreover, the heart in diabetic mice is characterized by TG accumulation and high turnover rates [6], whereas a decrease in myocardial TG content in heart failure patients was associated with higher levels of toxic lipid intermediates, such as diacylglycerol (DAG) and ceramides [7]. Diacylglycerol and ceramides are produced during TG synthesis and decomposition and are believed to be involved in lipid toxicity [8], impairments in the transcriptional regulation of metabolic gene expression in failing hearts, and a decrease in mitochondrial function, causing disturbances in energy supply for the contractile apparatus [1,9,10]. As lipid second messengers, DAG activates several isoforms of protein kinase C that have been linked to the development of pathological heart conditions, including ischemia, hypertrophic or dilated cardiomyopathy, diabetic cardiomyopathy, fibrosis, and inflammation [11].

Signaling pathways that are involved in lipid metabolism are committed to the pathogenesis and progression of various types of cardiac dysfunction. The stimulation of lipogenesis, followed by excessive intramyocardial fat accumulation, either in the presence of high-fat and/or carbohydrate intake, results in cardiomyopathy and coronary heart disease [12]. Chronic elevations of fatty acids (FAs) that derive from de novo lipogenesis (i.e., palmitic acid [16:0], palmitoleic acid [16:1n-7], and oleic acid [18:1n-9]) are associated with the development of coronary heart disease, incident heart failure, and sudden cardiac arrest [13]. Activation of the lipogenic factor sterol regulatory element-binding protein 1 (SREBP1) has been shown to lead to lipid accumulation within cardiomyocytes, resulting in cardiomyocyte dysfunction, and this effect was associated with peroxisome proliferator-activated receptor γ (PPARγ) activation [14]. The lipolysis of intracellular TGs by adipose triglyceride lipase (ATGL) also generates lipid ligands for PPARγ activation, which is known to induce target gene expression for FA uptake, β-oxidation enzymes, and both TG synthesis and lipolysis [10]. Inducible ATGL knockout in adult cardiomyocytes leads to heart steatosis and worsens heart function [15,16]. Heart-specific ATGL overexpression improves systolic function in the basal state. It protects the heart from the development of pressure-induced, high-fat diet-induced, doxorubicin-induced, and diabetic heart failure. The preservation of heart function in these mice is associated with a decrease in PPARα/peroxisome proliferator-activated receptor γ coactivator 1α (PGC1α) signaling pathway activity and FA oxidation rate and an increase in the rate of glucose oxidation to sustain adenosine triphosphate (ATP) production [17,18].

The rate of myocardial FA oxidation decreases with progression of the severity of heart failure [2]. Fatty acid oxidation is much more severely impaired than FA esterification in the heart in spontaneously hypertensive rats (SHRs), a genetic model of primary hypertension and pathological LV hypertrophy [19]. As hypertrophy develops with age, the SHR heart changes to a glycolytic/glucose-oxidative phenotype rather than a FA-oxidative phenotype [20], which is associated with a higher level of TGs [21], a decrease in PPARγ and carnitine palmitoyltransferase 1 (CPT1) expression [22], and a decrease in coronary endothelial lipoprotein lipase activity [23]. Interestingly, the substrate-mediated stimulation of FA oxidation prevents progressive cardiac remodeling in SHRs [24]. Moreover, the development of hypertension in SHRs is associated with dyslipidemia and insulin resistance [19]. An increase in DAG content in SHR hearts during early stages was suggested to be related to the initiation of cardiac hypertrophy before the development of hypertension [23,25]. Furthermore, *Plzf* gene has been suggested to play important role in the regulation of metabolic pathways, including PPAR signaling and cell cycle regulation, in SHRs [26]. Polyunsaturated fatty acid prevents heart failure by, among other things, regulating mitochondria [27]. In SHRs, long-term administration of the enriched with bioactive compounds a virgin olive oil exerts cardioprotective effect through reduction of plasma levels of Angiotensin II and cholesterol and decreased oxidative stress [28].

The above information underscores the important role of lipids and pathways that are involved in their synthesis and degradation in cardiomyocyte metabolism regulation and the development of cardiac dysfunction in various pathological states. However, exact role of lipids, their derivatives, and related metabolic pathways in the development of cardiac hypertrophy in SHRs is unknown. Thus, we used 6-week-old SHRs (SHR6 group) with the early development of hypertension and 18-week-old SHRs (SHR18 group) that are characterized by concentric hypertrophy with high blood pressure [20]. We determined the impact of disturbances of processes that are related to lipid metabolism (i.e., lipogenesis, lipolysis, and FA oxidation) in the development of pathological LV hypertrophy in SHRs with age. Our results showed that the progression of LV hypertrophy and heart dysfunction in SHRs was associated with TG accumulation, which was related to an increase in the expression of genes that are involved in their synthesis early in development and a decrease in the rate of lipolysis and β-oxidation of FA in cardiomyocytes.

## 2. Materials and Methods

### 2.1. Materials

α/β-Hydrolase domain containing 5 (ABDH5; 36A, catalog no. sc-100468), 5′-adenosine monophosphate-activated protein kinase (AMPK; H-300, catalog no. sc-25792), atrial natriuretic peptide (ANP; FL-153, catalog no. sc-20158), CPT1 (A-14, catalog no. sc-31128), elongation of very long chain fatty acids protein 1 (ELOVL1; H-65, catalog no. sc-135058), fatty acid transport protein 1 (FATP1; I20, sc-4497), G0/G1 switch protein 2 (G0S2; G-12, catalog no. sc-133423), phosphoinositide-dependent kinase-1 (PDK1; C-20, catalog no. sc-7140), PPARα (H-2, catalog no. sc-398394), stearoyl-CoA desaturase 1 (SCD1; E-15, catalog no. sc-14720), and SREBP1 (2A4, catalog no. sc-13551) antibodies were obtained from Santa Cruz Biotechnology (Santa Cruz, CA, USA). Protein kinase B (AKT; C73H10, catalog no. 2938), ATGL (catalog no. 2138), acetyl-CoA carboxylase (ACC; catalog no. 3662), extracellular signal-regulated kinase 1/2 (ERK1/2; L34F12, catalog no. 4696), phosphorylated AKT at Ser473 (pAKT[Ser473]; catalog no. 9271), pAKT at Thr308 (pAkt[Thr308]; catalog no. 9275), phosphorylated AMPK at Thr172 (pAMPK; catalog no. 2531), phosphorylated ERK1/2 at Thr202/Tyr204 (pERK1/2; 197G2, catalog no. 4377), S6 kinase (S6K; catalog no. 9202), and phosphorylated S6K at Thr389 (pS6K; catalog no. 9205) antibodies were obtained from Cell Signaling Technology (Hartsfordshire, UK). Oxidative phosphorylation system (OXPHOS; catalog no. ab110413), PGC1α (catalog no. ab54481), and thyroid receptor α (TRα; catalog no. ab53729) antibodies were obtained from Abcam (Cambridge, UK). Glyceraldehyde-3-phosphate dehydrogenase (GAPDH; 6C5, catalog no. MAB374), phosphorylated ACC at Ser79 (pACC; catalog no. 07-303), and sirtuin 1 (SIRT1; catalog no. 07-303) antibodies were obtained from Merck (Darmstadt, Germany). ELOVL5 (catalog no. SAB2100679) and ELOVL6 (catalog no. PRS4571) antibodies were obtained from Sigma (St. Louis, MO, USA). The other chemicals were purchased from Sigma unless otherwise specified.

### 2.2. Animals

Male SHRs and Wistar Kyoto (WKY) rats were obtained from the Department of Animal Breeding of the Medical University of Warsaw. The animals were housed in a pathogen-free facility at room temperature under a 12 h/12 h light/dark cycle. All of the animals were allowed ad libitum access to water and standard pelleted rat chow. The animals were euthanized at 6 weeks (WKY6 and SHR6 groups) or 18 weeks (WKY18 and SHR18 groups) of age. All of the protocols that were used in the present study were approved by the First Local Ethical Committee for Animal Experiments in Warsaw.

### 2.3. Blood and Tissue Sampling

The rats were fasted for 16 h and sacrificed. Blood was collected aseptically by direct cardiac puncture and centrifuged at 3000× *g* at 4 °C for 5 min to collect plasma (samples were aliquoted and stored at −80 °C). The heart was excised and weighed, and the LV was frozen in liquid nitrogen and stored at −80 °C.

Plasma TG and cholesterol levels were measured using commercial kits (BioSystems, Barcelona, Spain). Plasma free FA (FFA) levels were measured using the NEFA-HR(2) Kit (Wako, Richmond, VA, USA). Glucose levels were measured in blood samples that were collected from the tail using glucose strips with an Optium Xido glucose meter (Abbott, Alameda, CA, USA).

### 2.4. Echocardiography

Left ventricular function was evaluated using MyLab25 (Esaote) with a 13-MHz linear array transducer. Each rat was examined at 5 weeks (WKY6 and SHR6 groups) or 17 weeks (WKY18 and SHR18 groups) of age. Under light anesthesia (1.5–2.0% isoflurane by mask inhalation), LV end-diastolic and end-systolic diameters and wall thickness were determined from the short-axis view at the midpapillary level. Left ventricle end-diastolic and end-systolic diameters were planimetered from the parasternal long-axis view. Left ventricle ejection fraction was calculated as the following: (LV diastolic volume − LV systolic volume)/LV diastolic volume. Fractional shortening was calculated as the following: (LV diameter in diastole − LV diameter in systole)/LV diameter in diastole.

### 2.5. Gene Expression Analysis

Total RNA was isolated from the LV using Total RNA Mini Plus (A&A Biotechnology, Gdynia, Poland) according to the manufacturer’s instructions. DNase-treated RNA (A&A Biotechnology, Gdynia, Poland) was reverse-transcribed using the RevertAid H Minus First Stand cDNA Synthesis Kit (Thermo Scientific, Pittsburgh, PA, USA). Real-time quantitative polymerase chain reaction (PCR) was performed using the CFX Connect Real-Time PCR Detection System (Bio-Rad, Hercules, CA, USA). SsoAdv Univer SYBR SMX (Bio-Rad, Hercules, CA, USA) was used to detect and quantify mRNA expression. The relative expression of each sample was determined after normalization to *β-actin* or 60S ribosomal protein L32 (*Rpl32*) using the ΔΔCt method. A list of primers for real-time PCR is presented in Supplementary Table S1.

### 2.6. Western Blot Analysis

Left ventricle samples from WKY and SHR rats were homogenized in ice-cold buffer that contained 20 mM Tris-HCl (pH 7.4), 2 mM ethylene glycol-bis(β-aminoethyl ether)-*N,N,N′,N′*-tetraacetic acid (EGTA), 2 mM ethylenediamine tetraacetic acid (EDTA), 2 mM Na_3_VO_4_, 1 mM phenylmethylsulfonyl fluoride, 10 mM β-mercaptoethanol, 10 μg/μL leupeptin, 5 μg/μL pepstatin A, and 2 μg/μL aprotinin and centrifuged at 10,000× *g* for 20 min at 4 °C. The protein content in lysates was determined using the Bio-Rad Protein Assay (Bio-Rad) with bovine serum albumin as the reference. The proteins samples were separated on 10% sodium dodecyl sulfate-polyacrylamide gel electrophoresis gels and transferred to polyvinylidene difluoride membranes (Millipore, Billerica, MA, USA). Western blot analysis was performed using appropriate antibodies. The proteins were visualized using SuperSignal West Pico PLUS Chemiluminescent Substrate (Thermo Scientific) and quantified by densitometry. Eight samples from different experimental conditions were loaded on the same gel. Due to the limited amount of material for the study LV form 5 rats were merged in one sample and 3 different gels were run. Protein levels are expressed relative to the abundance of GAPDH. Phosphorylated protein levels are expressed relative to abundance of the unphosphorylated isoform of the respective protein.

### 2.7. Measurement of Lipids

Lipids were extracted using the method of Bligh and Dyer [29] and measured as described previously [30]. Briefly, the lipids were separated by thin-layer chromatography on silica gel-60 plates (Merck) in heptane/isopropyl ether/glacial acetic acid (60/40/4, vol/vol/vol) with authentic standards. Bands that corresponded to TG, FFA, DAG, and phospholipid standards were scraped off the plate and transferred to screw-cap glass tubes that contained methylpentadecanoic acid as an internal standard. Fatty acids were then transmethylated in the presence of 14% boron trifluoride in methanol. The resulting methyl esters were extracted with hexane and analyzed by gas-liquid chromatography. Total lipid contents were calculated from the individual FA content in each fraction.

### 2.8. Desaturation and Elongation Indices

The content of oleic (18:1n9) and 18:0 acids in total lipid extracts was analyzed by gas-liquid chromatography as described above and used to calculate the 18:1n9/18:0 ratio. The content of 18:0 and 16:0 was used to calculate the elongation index (18:0/16:0 ratio).

### 2.9. Statistical Analysis

The data are expressed as mean ± SD, with *n* = 10 rats/group. Multiple comparisons were performed using one-way analysis of variance (ANOVA) followed by Tukey’s post hoc test using Prism 8.3.0 software (GraphPad, La Jolla, CA, USA). A two-sided *t*-test was applied when differences between two groups were analyzed. The level of significance was *p* < 0.05.

## 3. Results

### 3.1. Plasma Parameters

The SHR6 group exhibited significant decrease in glucose, and increases in cholesterol, FFA, and TG levels compared with the WKY6 group (Table 1). The SHR18 group exhibited a decrease in glucose and cholesterol, and an increase in TG levels, whereas FFA levels were unchanged relative to the WKY18 group (Table 1).

### 3.2. Myocardial Structure and Function

The ratio of heart weight to body weight was significantly higher in both the SHR6 and SHR18 groups compared with the WKY control groups (Table 2). The SHR6 group exhibited a significantly higher heart rate compared with the WKY6 group (Table 2). The SHR6 group exhibited no change in cardiac structure or function relative to the WKY6 control group, except for a significant increase in relative LV wall thickness (RWT; Table 2). The SHR18 group exhibited an increase in heart rate compared with the corresponding WKY18 control group (Table 2). The SHR18 group exhibited a significant increase in anterior and posterior LV wall thickness in systole and diastole and RWT. The LV end-diastolic diameter was not significantly different from the control group, in contrast to end-systolic diameter, which was significantly higher. No change in end-systolic volume was found, but LV end-systolic volume was significantly higher than in the control group. Both, the LV ejection fraction and the systolic fraction were significantly lower in the SHR18 group relative to the WKY18 control group (Table 2).

### 3.3. Molecular Pathways of Myocardial Remodeling in SHRs

#### 3.3.1. Molecular Indicators of Myocardial Remodeling

**Figure 1:**
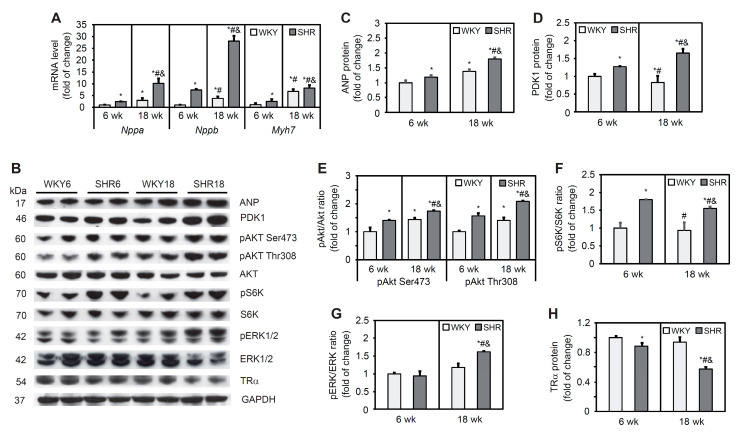
Molecular signaling pathways of cardiac remodeling in SHRs. *Nppa*, *Nppb*, and *Myh7* mRNA levels (**A**) were measured by real-time polymerase chain reaction. Atrial natriuretic peptide (ANP) (**B**,**C**), phosphoinositide-dependent kinase-1 (PDK1) (**B**,**D**), phosphorylated protein kinase B (pAKT) (**B**,**E**), phosphorylated S6 kinase (pS6K) (**B**,**F**), phosphorylated extracellular signal-regulated kinase 1/2 (pERK1/2) (**B**,**G**), and thyroid receptor α (TRα) (**B**,**H**) protein levels were determined by Western blot. The results are expressed as mean ± SD. *n* = 10; in the case of Western blot analysis LV form 5 rats were merged in one sample and 3 different gels were run. * *p* < 0.05, vs. WKY6 group; ^#^ *p* < 0.05, vs. SHR6 group; ^&^ *p* < 0.05, vs. WKY18 group.

Expression levels of the *Nppa* and *Nppb* genes, indicators of pathological LV myocardial hypertrophy, and the *Myh7* gene, an indicator of myocardial dysfunction, were significantly higher in SHRs compared with WKY rats in both age groups. In both SHRs and WKY rats, these values increased significantly with age (Figure 1A). The content of ANP protein, encoded by the *Nppa* gene, was similar in SHRs and WKY rats at 6 weeks of age. However, in 18-week-old rats, a 29% increase in ANP protein content was observed in SHRs compared with WKY rats (Figure 1B,C).

#### 3.3.2. AKT Pathway

AKT signaling plays a key role in regulating many cardiac physiological functions by regulating cardiomyocyte size, survival, angiogenesis, and inflammation in both the physiological and pathological LV [31]. PDK1 activates AKT through its phosphorylation at threonine 308. PDK1 protein content was higher in SHRs at 6 and 18 weeks of age relative to the respective control groups and increased with age. A decrease in PDK1 protein content with age was observed in the WKY group (Figure 1B,D). The level of AKT phosphorylation at serine 473 and threonine 308 was higher in SHRs relative to WKY rats in both age groups (Figure 1B,E). AKT, through the activation of mechanistic/mammalian target of rapamycin, phosphorylates and activates S6K. Levels of the phosphorylated form of S6K did not change in WKY rats with age, whereas it significantly increased in SHRs relative to the corresponding WKY age groups (Figure 1B,F). Overall, the AKT pathway was activated in 6- and 18-week-old SHRs, indicated by an increase in S6K kinase phosphorylation.

#### 3.3.3. Other Signaling Pathways Involved in the Development of Cardiac Hypertrophy

ERK1/2 is known to be activated in response to almost any hypertrophic stimulus that is induced by stress and agonists. The level of ERK1/2 phosphorylation was similar in both groups of 6-week-old animals, whereas it increased significantly in the SHR18 group compared with the respective WKY control group (Figure 1B,G). Genomic effects of thyroid hormone in the heart are mediated by nuclear TRs, and a decrease in TR levels occurs in pressure overload-induced cardiac hypertrophy [32]. TRα is the predominant isoform in the heart. TRα receptor protein content was lower in SHRs relative to WKY rats, and this decrease progressed with age (Figure 1B,H).

### 3.4. Lipid Content in the Heart

**Figure 2:**
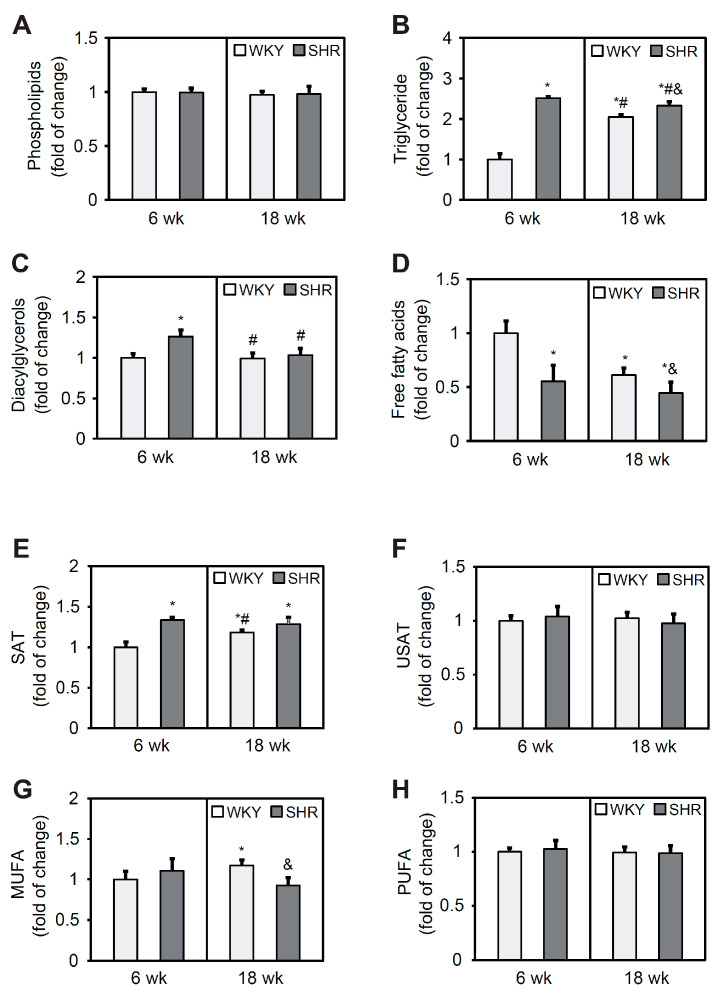
Phospholipid (**A**), triglyceride (**B**), diacylglycerol (**C**), free fatty acid (**D**), saturated fatty acid (SAT) (**E**), unsaturated fatty acid (USAT) (**F**), monounsaturated fatty acid (MUFA) (**G**), and polyunsaturated fatty acid (PUFA) (**H**) content in the left ventricle in WKY rats and SHRs at 6 and 18 weeks of age. Lipids were extracted from the heart, separated by thin-layer chromatography, and quantified by gas-liquid chromatography. The results are expressed as mean ± SD. *n* = 10. * *p* < 0.05, vs. WKY6 group; ^#^ *p* < 0.05, vs. SHR6 group; ^&^ *p* < 0.05, vs. WKY18 group.

Phospholipid content did not differ between groups at either 6 or 18 weeks of age (Figure 2A). Triglyceride levels were 2.5-fold higher in the SHR6 group compared with the WKY6 group. In 18-week-old animals, TG content in the SHR group was 14% higher than in WKY rats. In the WKY18 group, a two-fold increase in TG content was observed in cardiomyocytes compared with the WKY6 group. In contrast, in the SHR18 group, TG levels decreased by 8% relative to the SHR6 group (Figure 2B). The SHR6 group exhibited an increase in DAG content relative to the WKY6 group (Figure 2C). Free fatty acid content was lower in both the SHR6 and SHR18 groups relative to the respective WKY controls (by 45% and 29%, respectively). A significant 39% decrease in FFA levels occurred with age only in WKY rats (Figure 2D).

The FA composition analysis showed a 34% increase in saturated FA content in the SHR6 group relative to the WKY6 group. In WKY rats, saturated FA content increased with age, which was not observed in SHRs (Figure 2E). Unsaturated FA content did not change in either group (Figure 2F). Monounsaturated FA content was 10% higher in the SHR6 group compared with the WKY6 group and 22% lower in the SHR18 group compared with the WKY18 group (Figure 2G). The content of polyunsaturated FAs did not change in any of the groups (Figure 2H).

### 3.5. Lipogenesis Pathway in Cardiomyocytes

**Figure 3:**
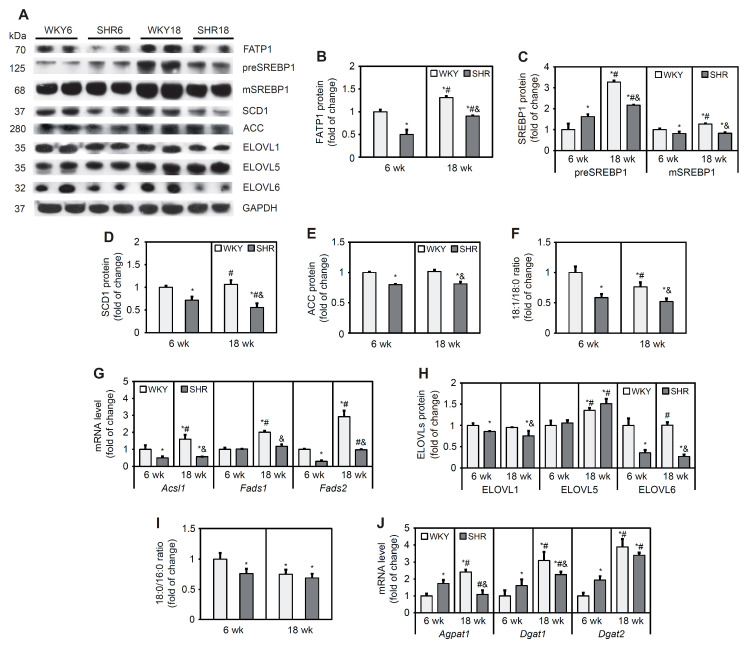
Expression of lipogenic factors and desaturation (18:1n-9/18:0) and elongation (18:1n9/18:0) ratios in cardiomyocytes in WKY rats and SHRs at 6 and 18 weeks of age. Protein levels of fatty acid transport protein 1 (FATP1) (**A**,**B**), premature and mature forms of sterol regulatory element-binding protein 1 (preSREBP1 and mSREBP1, respectively) (**A**,**C**), stearoyl-CoA desaturase 1 (SCD1) (**A**,**D**), acetyl-CoA carboxylase (ACC) (**A**,**E**), and elongases (ELOVLs) (**A**,**H**) were determined by Western blot. *Acsl1*, *Fads1*, *Fads2*, *Agpat1*, *Dgat1*, and *Dgat2* mRNA levels (**G**) were measured by real-time PCR. Palmitate, stearate, and oleate levels in cardiac total lipid extracts were measured by gas–liquid chromatography (**F**,**I**). The results are expressed as mean ± SD. *n* = 10; (**J**) in the case of Western blot analysis LV form 5 rats were merged in one sample and 3 different gels were run. * *p* < 0.05, vs. WKY6 group; ^#^ *p* < 0.05, vs. SHR6 group; ^&^ *p* < 0.05, vs. WKY18 group.

FATP1 is an integral membrane protein that is able to enhance FA uptake [33]. FATP1 protein content was 50% lower in the SHR6 group compared with the corresponding control group. In the SHR18 group, FATP1 protein content was 32% lower compared with the WKY18 group. In both groups, a significant increase in FATP1 levels was observed with age (by 31% in WKY rats and 80% in SHRs; Figure 3A,B).

SREBPs are initially synthesized in the endoplasmic reticulum as full-length precursor proteins. Upon activation, full-length precursor proteins are cleaved and then translocate to the nucleus and stimulate lipogenic gene transcription [34]. The SHR6 group exhibited an increase in levels of the precursor and a decrease in levels of the mature form of SREBP1 protein relative to the WKY6 group. The SHR18 group exhibited a lower content of both the precursor and mature forms of SREBP1 relative to the WKY18 group. In both the WKY and SHR groups, the content of the precursor form of SREBP1 increased with age (Figure 3A,C). The contents of target proteins of the transcription factor SREBP1 that are involved in FA synthesis (i.e., SCD1 and ACC) decreased in the SHR6 group (by 27% for SCD1 and 20% for ACC) compared with the respective control group. SCD1 and ACC protein contents significantly decreased by 49% and 19%, respectively, in the SHR18 group compared with the WKY18 group (Figure 3A,D,E). The desaturation index (an indicator of SCD1 activity) was lower in SHRs than in WKY rats in both age groups and decreased with age in both groups (Figure 3F).

Expression of the *Acsl1* gene, which encodes acetyl-coenzyme A (CoA) synthetase, was downregulated in SHRs compared with WKY rats at 6 and 18 weeks of age (Figure 3G). *Fads1* and *Fads2* encode the rate-limiting ∆5 and ∆6 desaturase enzymes, respectively, in the polyunsaturated fatty acid biosynthesis pathway [35]. The expression level of *Fads1* was unchanged in the SHR6 group, whereas the mRNA level of *Fads2* decreased in this group compared with the WKY6 group. In the SHR18 group, the levels of both *Fads1* and *Fads2* were lower compared with WKY controls (Figure 3G).

ELOVL catalyzes the first, rate-limiting step in the cycle that adds two carbons to acyl chains of FAs, with 12 or more carbons per chain [36]. ELOVL1 and ELOVL6 protein content significantly decreased in SHRs relative to WKY rats (Figure 3A,H). ELOVL5 protein content was similar in 6- and 18-week-old WKY rats and SHRs. ELOVL5 content increased in both groups with age (Figure 3A,H). The elongation index (i.e., an indicator of elongase activity) decreased in the SHR6 group and remained unchanged in the SHR18. In cardiomyocytes in WKY rats, the elongation index decreased with age but did not change in the SHR group (Figure 3I).

Expression of the *Agpat1*, *Dgat1*, and *Dgat2* genes, which encode enzymes that are involved in TG synthesis, were higher in the SHR6 group and decreased in the SHR18 group relative to the respective WKY control groups (Figure 3J).

In summary, the expression of genes that encode de novo FA synthesis proteins and the content of FA synthesis proteins were downregulated in cardiomyocytes in the SHR6 and SHR18 groups compared with respective WKY controls. This suggests a lower rate of de novo FA synthesis in these animals. The expression level of genes that are involved in TG synthesis was elevated in the SHR6 group as opposed to the SHR18 group, which may explain the high TG content in these animals.

### 3.6. Fatty Acid β-Oxidation

**Figure 4:**
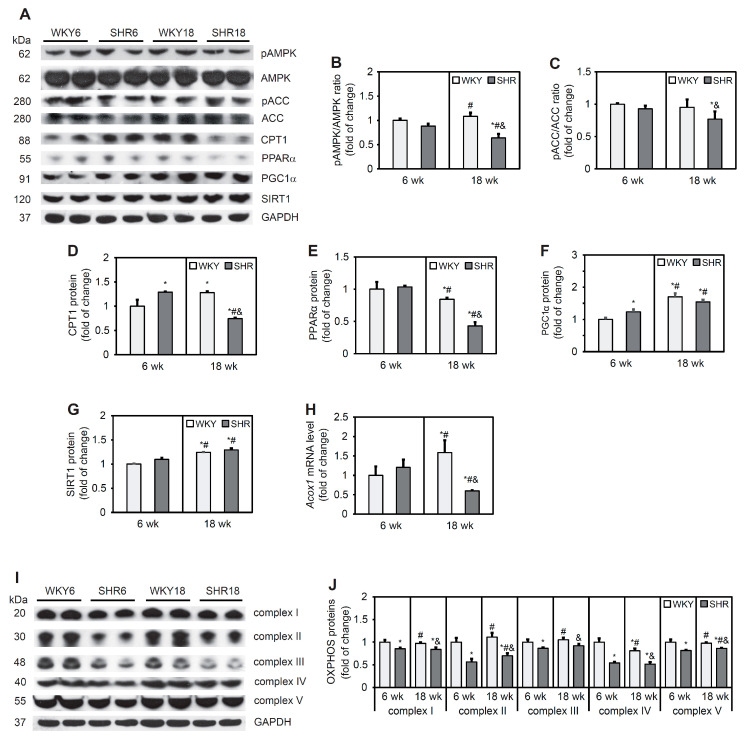
Expression of proteins that are involved in fatty acid oxidation and proteins of respiratory chain complexes. AMP-activated protein kinase (AMPK) (**A**,**B**), acetyl-CoA carboxylase (ACC) (**A**,**C**), carnitine palmitoyltransferase 1 (CPT1) (**A**,**D**), peroxisome proliferator-activated receptor α (PPARα) (**A**,**E**), peroxisome proliferator-activated receptor γ coactivator 1 α (PGC1α) (**A**,**F**), sirtuin 1 (SIRT1) (**A**,**G**), and OXPHOS (**I**,**J**) protein levels and phosphorylation of AMPK (**A**,**B**) and ACC (**A**,**C**) were determined by Western blot analysis. Acox1 mRNA levels (**H**) were measured by real-time PCR. The results are expressed as mean ± SD. *n* = 10; in the case of Western blot analysis LV form 5 rats were merged in one sample and 3 different gels were run. * *p* < 0.05, vs. WKY6 group; ^#^ *p* < 0.05, vs. SHR6 group; ^&^ *p* < 0.05, vs. WKY18 group.

Fatty acid oxidation has been shown to be modulated by the activity of AMPK, which is activated by phosphorylation at a threonine residue. The SHR18 group exhibited lower levels of AMPK and lower phosphorylation of its downstream target, ACC, compared with the WKY18 group (Figure 4A–C). CPT1 protein content in the SHR6 group was 29% higher than in the WKY6 group. At 18 weeks of age, however, a 42% decrease in CPT1 protein content was observed in SHRs compared with WKY rats (Figure 4A,D).

PPARα is a transcription factor that is responsible for transcriptionally regulating genes that are associated with FA oxidation. The protein content of PPARα was similar in 6-week-old animals but lower in the SHR18 group relative to the corresponding WKY18 control group (Figure 4A,E). In contrast, content of the PPARα coactivator protein PGC1α was higher in the SHR6 group relative to the WKY6 group, whereas no change in the content of this protein was observed in 18-week-old animals. PGC1α protein content increased with age in both groups (Figure 4A,F). Similar trends were observed for SIRT1, which can deacetylate PGC-1α and thus upregulate its transcriptional function [37] (Figure 4A,G). An increase in expression level of the *Acox1* gene, which encodes acyl-CoA oxidase 1 protein, was observed in the WKY18 group compared with the WKY6 group. The SHR18 group exhibited lower *Acox1* expression compared with 6-week-old animals. In the SHR6 group, no change in *Acox1* expression was found compared with the WKY6 control group (Figure 4H).

Changes in PGC-1α/SIRT1 signaling affect mitochondrial homeostasis. Western blot analysis showed a lower protein content of respiratory chain complexes in SHRs at both 6 and 18 weeks of age (complex I by 15% and 16%, complex II by 44% and 30%, complex III by 14% and 8%, complex IV by 46% and 48%, and complex V by 18% and 14%, respectively) relative to the respective WKY6 and WKY18 control groups (Figure 4I,J).

In conclusion, a decrease in AMPK phosphorylation and PPARα protein content and the expression of its target gene, *Acox1***,** was observed in SHR rats at 18 weeks of age, suggesting a decrease in the rate of FA β-oxidation in cardiomyocytes. This was reflected by a significant decrease in all respiratory chain complex proteins in SHRs compared with WKY rats.

### 3.7. Lipolysis Process in Cardiomyocytes in SHRs

**Figure 5:**
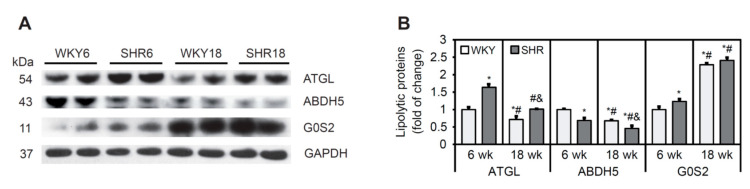
The process of lipolysis in cardiomyocytes in SHRs. (**A**,**B**) Protein levels of adipose triglyceride lipase (ATGL), α/β-hydrolase domain containing 5 (ABDH5), and G0/G1 switch protein 2 (G0S2) were determined by Western blot. The results are expressed as mean ± SD. *n* = 10; LV form 5 rats were merged in one sample and 3 different gels were run. * *p* < 0.05, vs. WKY6 group; ^#^ *p* < 0.05, vs. SHR6 group; ^&^ *p* < 0.05, vs. WKY18 group.

The rate-limiting step for TG hydrolysis is mediated by ATGL. G0S2 protein inhibits ATGL, whereas ABDH5 enhances the lipolytic activity of ATGL [38]. The protein content of ATGL was significantly higher in SHRs at both 6 and 18 weeks of age compared with WKY rats (Figure 5A,B). The protein content of ABDH5 was lower in SHRs than in WKY rats at both ages. The levels of ATGL and ABDH5 decreased in WKY rats and SHRs with increasing age (Figure 5A,B). The protein content of G0S2 was higher in the SHR6 group than in WKY rats, whereas no such change was observed at 18 weeks of age. The protein content of G0S2 increased significantly with age in both SHRs and WKY rats (Figure 5A,B).

In conclusion, SHR rats exhibited a decrease in protein content of the ATGL activator ABDH5 and an increase in the content of its inhibitor, G0S2, indicating that the rate of lipolysis was lower in SHRs at 6 and 18 weeks of age, which was associated with higher TAG in cardiomyocytes.

## 4. Discussion

Heart-to-body weight ratio was increased in six-week-old SHRs compared with six-week-old WKY rats. Moreover, significantly higher RWT and the expression of genes that encode fetal proteins (*Nppa* and *Nppb*), myosin heavy chains (*Myh7*), and ANP protein content indicates the activation of mechanisms that lead to cardiac dysfunction. Eighteen-week-old SHRs were characterized by LV hypertrophy, indicated by a higher heart-to-body weight ratio and results of the echocardiographic analysis. They also had impaired LV systolic function as evidenced by the reduction of the ejection and the shortening fractions. These changes in cardiac function and structure have been associated with alterations of lipid metabolism in cardiomyocytes. We observed higher levels of TGs in cardiomyocytes in the SHR6 and SHR18 groups. Lipid accumulation in the heart has been shown to be associated with cardiac dysfunction in obese Zucker diabetic fatty rats [39], and TAG turnover rate decreased in pressure-overloaded failing hearts [7]. Changes in cardiac energy metabolism occur within 4–8 weeks during the development of cardiac hypertrophy [21,40,41]. Our results suggest that the disruption of lipid metabolism in 6-week-old SHRs may contribute to the development of heart hypertrophy with age. This was confirmed by the accumulation of DAG in the heart in the SHR6 group, in which an increase in DAG content in SHR hearts during early stages appears to be related to the initiation of cardiac hypertrophy in SHR hearts before hypertension develops [25].

Chronic elevations of FAs, especially saturated FAs, that we observed in cardiomyocytes in the SHR6 group are related to the development of heart failure [13]. Interestingly, levels of proteins that are involved in the transcriptional control of lipogenesis (SREBP1) and de novo FA synthesis (ACC) decreased in SHRs at both 6 and 18 weeks of age. We also observed a decrease in the expression of genes that encode FA turnover proteins (*Acsl1*, *Fads1*, and *Fads2*). Furthermore, the protein content of SCD1, ELOVL1, and ELOVL6 and consequently the desaturation and elongation indices also decreased. These results and the decrease in FFA content indicate a lower rate of de novo FA synthesis in SHR cardiomyocytes.

Factors that regulate lipogenesis in cardiomyocytes are also involved in the regulation of myocardial remodeling. SREBP1 controls the expression of lipogenic genes, such as *Scd1* and *Acc*. SREBP1c levels were markedly higher in LV biopsies from patients with metabolic syndrome. This effect was followed by a lower LV ejection fraction and the greater intracellular accumulation of lipids in cardiomyocytes [14]. Moreover, alterations of SREBP1 activity may contribute to the development of ventricular arrhythmias [42]. SREBP1c is regulated through phosphorylation by mitogen-activated protein kinases ERK1/2 [43]. SHRs at 18 weeks of age exhibited a significant increase in the activating phosphorylation of ERK1/2. Similarly, the AKT pathway is involved in the activation of lipogenesis [31], the upregulation of which was observed in SHRs. However, these pathways did not increase de novo FA synthesis in SHR rats, indicating that the activation of these kinases was associated with the development of LV hypertrophy. This was also indicated by an increase in the activating phosphorylation of S6K in the heart in SHRs, which was previously shown to contribute to pathological cardiac remodeling [44] and by findings that the simultaneous activation of mitogen-activated protein kinase and AKT regulated key pathways that are crucial for the development of LV hypertrophy and cardiac dysfunction in SHRs [45].

Thyroid hormone exerts important cardiovascular effects, and abnormalities of its metabolism cause cardiovascular morbidity [32]. Thyroid hormone regulates the expression of numerous genes that are engaged in lipogenesis by binding to TRs. Furthermore, thyroid hormones indirectly control the transcriptional regulation of lipogenesis as a consequence of their effects on the expression and activity of SREBP1c that, in conjunction with TR, regulates ACC transcription [46]. We observed a decrease in protein levels of TRα in cardiomyocytes in SHRs, and this disturbance in the genomic TH pathway could possibly play a role in the development of cardiac dysfunction and impairments in lipid metabolism in the heart in SHRs. Increased TG content in cardiomyocytes could also be related to decreased leptin concentration in plasma in SHRs [47], because it was shown that rescue of cardiac leptin receptors in *db/db* mice prevents myocardial TG accumulation [48]. Moreover, it was suggested that novel hepatokine angiopoietin-like protein 8 is as factor that interact with leptin and protect cardiac remodeling among youths with risk for metabolic syndrome by modulation of plasma TG concentration [49,50]. However, contribution of TH and leptin in alternation of lipid metabolism and cardiac function in SHRs requires further research.

The other reason for the decrease in FFA content in cardiomyocytes in SHRs is also likely attributable to impairments in FA uptake. SHRs have a defect in the gene that encodes CD36 protein, resulting in the production of an inactive protein [19,51]. Our results showed that in the heart in SHRs, the protein content of another FA transporter, FATP1, decreased. The above changes resulted in a decrease in FFA content in cardiomyocytes, although plasma FFA levels were significantly higher in SHRs at 6 weeks of age and unchanged at 18 weeks of age compared with WKY controls. Activity of the transcription factor SREBP1 is regulated precisely by the level of FFA in cardiomyocytes [52]. The long-term lack of SREBP1 activation by FFAs not only resulted in a decrease in the rate of lipogenesis but could also affect the process of SREBP1 protein maturation [52]. Indeed, although an increase in the immature form of SREBP1 was observed in SHRs at 6 weeks of age, a decrease in both the mature and immature forms was observed in 18-week-old SHRs. These results indicate that impairments in FA uptake affect SREBP1 maturation and lipogenesis in the heart in SHRs.

Left ventricle hypertrophy is often associated with an increase in TG content in patients’ plasma [53]. In patients who were diagnosed with metabolic syndrome and hypertension, an increase in TG content was also observed in cardiomyocytes [14]. The present study showed that TG levels were elevated in plasma in SHRs compared with WKY rats, and an increase in TG content in cardiomyocytes occurred as early as 6 weeks of age in SHRs. Excessive TG accumulation in 6-week-old SHRs was associated with an increase in the expression of genes that encode proteins that are involved in TG synthesis (*Agpat1*, *Dgat1*, and *Dgat2*). The transgenic overexpression of DGAT1 doubled TG content in the heart [54]. Additionally, the overexpression of DGAT1 in the heart resulted in the development of cardiomyopathy, an increase in cardiac fibrosis, and a decrease in heart mitochondrial biogenesis over time [55]. Thus, the activation of genes that are involved in TG synthesis may contribute to the development of cardiac dysfunction in SHRs at an early age. However, in 18-week-old rats, the expression of TG synthesis genes was significantly reduced, although TG content in cardiomyocytes was still elevated. These results indicate that disruption of the activity of pathways that are involved in TG degradation likely occurs in cardiomyocytes in 18-week-old SHRs.

Indeed, the phosphorylation of proteins that are involved in FA β-oxidation (AMPK and ACC) decreased in the SHR18 group, suggesting a lower rate of this process. The content of CPT1 protein, which is the main protein that mediates the transport of FA to mitochondria, was higher in 6-week-old SHRs but decreased in 18-week-old SHRs relative to appropriate WKY controls. This supports the theory that in response to hypertrophy the SHR heart switches to a glycolytic/glucose-oxidative phenotype from predominantly FA oxidative metabolism [20]. The fact that a decrease in AMPK-dependent pathway activity may contribute to an increase in TG accumulation in the heart may be supported by a study in SHR-stroke prone rats that were supplemented with dietary AMP for 3 weeks. These rats exhibited decreases in plasma and liver levels of TGs, which was further associated with an increase in the expression of the gene that encodes AMPK protein [56]. Furthermore, it has been shown that AMPK activation in SHRs using metformin improves cardiac function and prevents LV hypertrophy [57]. Together, these results underline crucial role of AMPK in the development of functional and metabolic disturbances in SHRs.

A decrease in CPT1 protein in 18-week-old SHRs was related to a decrease in PPARα, PGC1α, and SIRT1 proteins and *Acox1* gene expression. The lower content and activity of proteins that regulate the rate of FA β-oxidation was reflected by a decrease in protein levels of all five respiratory chain complexes. This may indicate that the metabolic switch from FAs to other energy sources is unable to meet the energy requirements of SHR cardiomyocytes as suggested by Rubattu et al. [58]. A reduction of the rate of β-oxidation in favor of the activation of glucose oxidation is also associated with a mutation of the *Cd36* gene. In adipose tissue and muscle in SHRs, this was associated with excessive TG and DAG accumulation and a decrease in insulin sensitivity [19]. A growing body of evidence shows that an imbalance in FA oxidation contributes to the development of heart failure. Therefore, we hypothesized that the decrease in the activity of oxidative pathways that are regulated by AMPK and PPARα in 18-week-old SHRs may explain the higher TG levels in cardiomyocytes and associated myocardial dysfunction.

Excessive TG accumulation in cardiomyocytes may also be caused by a decrease in lipolysis [16]. Inducible ATGL knockout in adult cardiomyocytes leads to heart steatosis and worsens heart function [59]. In primary neonatal cardiomyocytes, ATGL silencing induces toxic ceramide synthesis and accumulation through the inhibition of FA oxidation [60]. Heart-specific ATGL overexpression protects the heart from the development of pressure-induced, high-fat diet-induced, doxorubicin-induced, and diabetic heart failure [61,62]. Although ATGL levels were elevated in the heart in SHRs, its activity likely decreased because this was associated with lower levels of the ATGL activator ABDH5 and higher levels of the ATGL inhibitor G0S2, and these changes were more pronounced in 18-week-old SHRs than in 6-week-old SHRs. Cardiac-specific ABHD5 deficiency leads to steatosis and heart failure through the inhibition of lipolysis and PPARα-dependent FA oxidation, which provokes endoplasmic reticulum stress and mitochondrial dysfunction [63,64]. Thus, the inhibition of ATGL-dependent lipolysis is another cause of TG accumulation that leads to a lower rate of FA β-oxidation in SHR cardiomyocytes.

## 5. Conclusions

**Figure 6:**
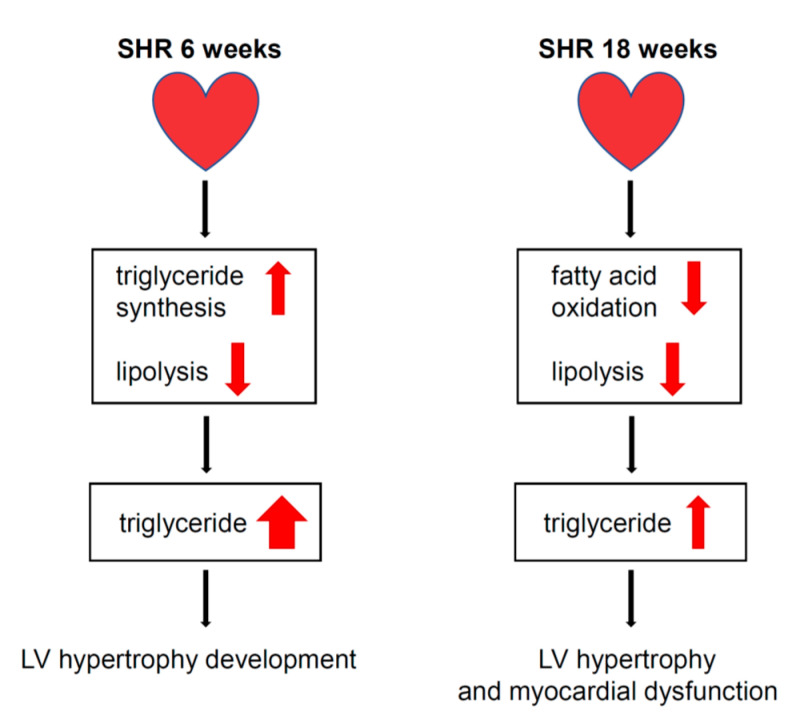
Proposed mechanism leading to cardiomyocyte steatosis, development of LV hypertrophy and myocardial dysfunction in SHRs. The progression of LV hypertrophy and myocardial dysfunction in the SHR6 group was associated with severe TG accumulation, which was associated with increased TG synthesis gene expression and decreased rate of lipolysis in cardiomyocytes. In the SHR18 group, LV hypertrophy and myocardial dysfunction were associated with TG accumulation, which could be attributed to a lower rate of lipolysis and β-oxidation in cardiomyocytes.

In the present study, we found that TG accumulation that was associated with congenital hypertension in SHRs resulted from decreases in lipolysis and FA β-oxidation in cardiomyocytes and was not associated with an increase in de novo FA synthesis. Lack of the activation of lipogenesis may be one of the mechanisms that leads to the development of pathological LV hypertrophy and consequently myocardial dysfunction that accompanies hypertension in SHRs (Figure 6).

## Figures and Tables

**Table 1:** Concentrations of plasma glucose, FFA, TG, and cholesterol in WKY and SHR rats at 6 and 18 weeks of age.

	WKY6	SHR6	WKY18	SHR18
Glucose (mg/dL)	182.9 ± 23.0	109.2 ± 19.9 *	213.9 ± 36.1	152.1 ± 24.1 ^&^
FFA (mg/dL)	4.9 ± 0.5	16.4 ± 5.5 *	9.7 ± 1.3	10.1 ± 2.0
TG (mg/dL)	73.7 ± 9.2	104.9 ± 23.2 *	86.3 ± 13.0	140.3 ± 24.6 ^&^
Cholesterol (mg/dL)	57.1 ± 5.0	66.6 ± 7.8 *	69.9 ± 11.3	37.8 ± 5.1 ^&^

*n* = 10. FFA—free fatty acid, TG—triglyceride. * *p* < 0.05, vs. WKY6 group; ^&^ *p* < 0.05, vs. WKY18 group.

**Table 2:** Echocardiographic analysis of heart function and structure in WKY and SHR rats at 6 and 18 weeks of age.

	WKY6	SHR6	WKY18	SHR18
HW/BW (g/g × 100)	0.38 ± 0.06	0.47 ± 0.07 *	0.29 ± 0.05	0.41 ± 0.03 ^&^
HR (beats/min)	312 ± 17	404 ± 26 *	351 ± 4	381 ± 26 ^&^
AWTd (mm)	1.14 ± 0.13	1.19 ± 0.11	1.60 ± 0.08	1.97 ± 0.14 ^&^
PWTd (mm)	1.24 ± 0.13	1.31 ± 0.11	1.68 ± 0.05	2.15 ± 0.15 ^&^
AWTs (mm)	2.46 ± 0.16	2.54 ± 0.21	3.18 ± 0.10	3.52 ± 0.26 ^&^
PWTs (mm)	2.73 ± 0.21	2.72 ± 0.16	3.33 ± 0.10	3.98 ± 0.20 ^&^
EDD (mm)	5.03 ± 0.60	4.79 ± 0.38	7.00 ± 0.24	6.83 ± 0.44
ESD (mm)	2.09 ± 0.37	2.10 ± 0.26	3.30 ± 0.26	3.83 ± 0.25 ^&^
RWT	0.50 ± 0.02	0.55 ± 0.03 *	0.48 ± 0.01	0.63 ± 0.03 ^&^
EDV (mL)	0.14 ± 0.05	0.12 ± 0.03	0.36 ± 0.04	0.34 ± 0.07
ESV (mL)	0.010 ± 0.006	0.010 ± 0.004	0.038 ± 0.010	0.06 ± 0.012 ^&^
EF (%)	92.5 ± 2.5	91.5 ± 1.8	89.5 ± 1.5	82.3 ± 1.3 ^&^
FS (%)	058 ± 0.05	0.56 ± 0.03	0.53 ± 0.02	0.44 ± 0.01 ^&^

*n* = 10. BW—body weight, HW—heart weight, HR—heart rate, AWTd—anterior wall thickness in diastole, PWTd—posterior wall thickness in diastole, AWTs—anterior wall thickness in systole, PWTs—posterior wall thickness in systole, EDD—end-diastolic diameter, ESD—end-systolic diameter, RWT—relative wall thickness, EDV—end-diastolic volume, ESV—end-systolic volume, EF—ejection fraction, FS—fractional shortening. * *p* < 0.05, vs. WKY6 group; ^&^ *p* < 0.05, vs. WKY18 group.

## Data Availability

The data that support the findings of this study are available from the corresponding author upon reasonable request.

## Supplementary Materials

The following supporting information can be downloaded at: https://www.mdpi.com/article/10.3390/cells11193032/s1, Table S1. Real-time PCR primers list.
