# Supplementary material for: Alterations of Lipid Metabolism in the Heart in Spontaneously Hypertensive Rats Precedes Left Ventricular Hypertrophy and Cardiac Dysfunction
Source: Cells. 2022 Sep 27;11(19):3032. doi: 10.3390/cells11193032 (PMC9563594; doi:10.3390/cells11193032)
Supplement: Supplementary file 1 [file cells-11-03032-s001.zip › cells-1883489-supplementary.pdf]

Supplementary Table S1. Real-time PCR primers list.

Gene	Forward Primer	Reverse Primer	Expected Size of the Amplicon	Annealing Temperatures	Accession Number
Acox1	TCGAAGCCAGCGTTATGAGG	GGGTCCGAGATGCCATATTCC	81 bp	60.18/60.34°C	NM_017340
Acsl1	GAGCAATGGTCACCCACCA	GGGAAGCGATGAATGCACTCT	81 bp	59.93/60.75°C	NM_012820
Agpat1	GCTAGGACATCCCCAAATCCTG	ACAGCTCCATTCTGGTCACCT	150 bp	60.49/61.11°C	NM_212458
Dgat1	CCAGTGGGTTCCCTGTTTGC	CCACAGATTGACATCCCGGT	81 bp	61.46/59.75°C	NM_053437
Dgat2	AGACCAAATTCGGCCTTCCA	CATTCCTTCCAGGAGCTGGC	81 bp	59.60/60.75°C	NM_001012345
Fads1	TGGAGAGCAACTGGTTTGTG	GTTGAAGGCTGACTGGTGAA	131 bp	58.32/58.03°C	NM_053445
Fads2	TGTCCACAAGTTTGTCATTGG	ACACGTGCAGGCTCTTTATG	128 bp	56.93/ 58.56°C	NM_031344
Myh7	GGACCCTGGAGGACCAGATG	GGTCAGCTGAGAGATAAGAG	158 bp	61.34/54.40°C	NM_017240
Nppa	ATCACCAAGGGCTTCTTCCT	GTGGTCTAGCAGGTTCTTGA	120 bp	58.62/56.87°C	NM_012612
Nppb	AATCCACGATGCAGAAGCTG	GCGCTGTCTTGAGACCTAAG	130 bp	58.62/58.37°C	NM_031545
Rpl32	GAAAGAGCAGCACAGCTGGC	TCATTCTCTTCGCTGCGTAGC	68 bp	62.20/60.80°C	NM_013226
β-actin	CTTGCAGCTCCTCCGTCGCC	CTTGCTCTGGGCCTCGTCGC	228 bp	65.85/65.85°C	NM_031144
